# A Truncated Form of Rod Photoreceptor PDE6 β-Subunit Causes Autosomal Dominant Congenital Stationary Night Blindness by Interfering with the Inhibitory Activity of the γ-Subunit

**DOI:** 10.1371/journal.pone.0095768

**Published:** 2014-04-23

**Authors:** Gaël Manes, Pallavi Cheguru, Anurima Majumder, Béatrice Bocquet, Audrey Sénéchal, Nikolai O. Artemyev, Christian P. Hamel, Philippe Brabet

**Affiliations:** 1 Inserm U1051, Institute for Neurosciences of Montpellier, Montpellier, France; 2 University of Montpellier 1, Montpellier, France; 3 University of Montpellier 2, Montpellier, France; 4 Department of Molecular Physiology and Biophysics, University of Iowa, Iowa City, Iowa, United States of America; 5 Department of Ophthalmology and Visual Sciences, University of Iowa, Iowa City, Iowa, United States of America; 6 CHRU, Genetics of Sensory Diseases, Montpellier, France; University of Oldenburg, Germany

## Abstract

Autosomal dominant congenital stationary night blindness (adCSNB) is caused by mutations in three genes of the rod phototransduction cascade, rhodopsin (*RHO*), transducin α-subunit (*GNAT1*), and cGMP phosphodiesterase type 6 β-subunit (*PDE6B*). In most cases, the constitutive activation of the phototransduction cascade is a prerequisite to cause adCSNB. The unique adCSNB-associated *PDE6B* mutation found in the Rambusch pedigree, the substitution p.His258Asn, leads to rod photoreceptors desensitization. Here, we report a three-generation French family with adCSNB harboring a novel *PDE6B* mutation, the duplication, c.928-9_940dup resulting in a tyrosine to cysteine substitution at codon 314, a frameshift, and a premature termination (p.Tyr314Cysfs*50). To understand the mechanism of the PDE6β1-314fs*50 mutant, we examined the properties of its PDE6-specific portion, PDE6β1-313. We found that PDE6β1-313 maintains the ability to bind noncatalytic cGMP and the inhibitory γ-subunit (Pγ), and interferes with the inhibition of normal PDE6αβ catalytic subunits by Pγ. Moreover, both truncated forms of the PDE6β protein, PDE6β1-313 and PDE6β1-314fs*50 expressed in rods of transgenic *X. laevis* are targeted to the phototransduction compartment. We hypothesize that in affected family members the p.Tyr314Cysfs*50 change results in the production of the truncated protein, which binds Pγ and causes constitutive activation of the phototransduction thus leading to the absence of rod adaptation.

## Introduction

Congenital stationary night blindness (CSNB; MIM 163500) is a clinically and genetically heterogeneous group of non-progressive hereditary retinal disorders characterized by night blindness and decreased visual acuity [1]. The mode of inheritance can be autosomal dominant, autosomal recessive, or X-linked. Mutations in rhodopsin (*RHO*), rod cGMP phosphodiesterase type 6 β-subunit (*PDE6B*), and rod transducin α-subunit (*GNAT1*), three genes of the rod phototransduction cascade, have been associated with the autosomal dominant CSNB (adCSNB) [2]–[4]. PDE6 mediates the phototransduction cascade from rhodopsin to cGMP-gated cation channels in the rod plasma membrane. PDE6 is a membrane-associated heterotetrameric enzyme composed of two large homologous catalytic subunits (PDE6α and PDE6β) and two identical small inhibitory subunits PDE6γ (Pγ). In the dark, the two Pγ subunits are bound to the PDE6αβ complex and inhibit its catalytic activity [5]–[7]. Upon light stimulation, rhodopsin activates its downstream target, transducin, which in turn binds the effector enzyme PDE6, resulting in the relief of the inhibition of the PDE6αβ catalytic subunits by the Pγ [8]–[10]. The activated PDE6αβ hydrolyzes intracellular cGMP thereby lowering its concentration and producing a closure of cGMP-gated channels and the hyperpolarization of the rod outer segment plasma membrane [8], [10], [11].

Mutations in the genes *PDE6A*, *PDE6B*, and *PDE6G*, encoding for the α-, β- and γ-subunits of PDE6 respectively, cause autosomal recessive retinitis pigmentosa (RP; MIM 268000), a degenerative retinopathy [12]–[14]. However, one single mutation in *PDE6B*, the substitution p.His258Asn, has been observed in the adCSNB condition [4]. Night blindness is an early symptom common to RP and CSNB resulting from functional dysfunction of rod photoreceptors. The loss of rod photoreceptor sensitivity is non-progressive in the CSNB condition whereas the rod photoreceptors death is observed simultaneously with the progressive impairment of the peripheral day vision in the RP condition. Eventually, the central vision is affected after several decades as cone degenerate in RP patients. No photoreceptor cell death is observed in patients with CSNB.

In this study, we report a new adCSNB-associated mutation. This mutation truncates PDE6β, but the truncated product keeps the ability to bind Pγ while being unable to hydrolyze cGMP. We show that the addition of the truncated PDE6β to normal PDE6αβ catalytic subunits interferes with the enzyme inhibition by Pγ, thus supporting the hypothesis that the mutant protein causes constitutive activation of the phototransduction, and hence rod desensitization.

## Materials and Methods

### Clinical examination

The participants provided their written informed consent by signing the declaration of permission for clinical examination and genetic analysis. All procedures were performed after obtaining specific approval for this study from the Montpellier University Hospital ethics committee, in agreement with the Declaration of Helsinki. Clinical examination included assessment of visual acuity, slit lamp biomicroscopy, direct funduscopy, autofluorescence imaging and electro-retinography (ERG) testing. ERG was performed in accordance with the standards of the International Society for Clinical Electrophysiology of Vision (ISCEV). Dark adaptometry was performed with a Goldman Weekers apparatus and the responses were recorded at 11° above fixation after 3 minutes of pigment bleaching.

### Mutation screening

Genomic DNA was isolated from 10 ml peripheral blood leucocytes using standard salting out procedure [15]. The DNA samples were quantified by spectrophotometry and diluted to 25 ng/µl for PCR amplification. Coding exons and adjacent intronic sequences of candidate genes were sequenced with an Applied Biosystems 3130xL genetic analyser (Applied Biosystems, Foster City, CA) using a BigDye Terminator cycle sequencing ready reaction kit V3.1 (Applied Biosystems, Foster City, CA) following manufacturer's instructions. Primer pairs and PCR conditions are available on request. Sequence analysis and mutation identification were performed using the Collection and Sequence Analysis software package (Applied Biosystems, Foster City, CA).

### Generation of transgenic X. laevis

cDNA coding PDE6β1-313 was PCR amplified using human retinal cDNA library and inserted into the pXOP (−508/+41)EGFP vector using *Not*I and *Xma*I sites [16]. cDNA coding PDE6β1-314fs*50 was PCR amplified from the pUC57-PDE6β1-314fs*50 template and cloned into the pXOP (−508/+41)EGFP vector using *Not*I and *Xma*I sites. The pXOP(−508/+41)EGFP-PDE6β1-313 and pXOP(-508/+41)EGFP- PDE6β1-314fs*50 plasmids were purified using Qiagen Miniprep or Midiprep with final elution in water (Qiagen, Venio, Netherlands). DNA sequencing of the constructs was performed to confirm the sequences. Purified plasmids were linearized with *Xho*I and subsequently used for microinjections. Experiments involving frogs were performed according to the protocol approved by the University of Iowa Animal Care and Use Committee. Transgenic tadpoles were produced by the restriction enzyme-mediated integration method [17]. Tadpoles were maintained at 18–22°C until two weeks and then sacrificed for further analysis.

### Live cell and cryosection imaging

Tadpoles expressing EGFP-PDE6β1-313 or EGFP- PDE6β1-314fs*50 in retinal rod cells were screened using an MZ16 Leica fluorescence microscope with GFP filter. For live imaging of retinal rod cells, transgenic tadpoles were anesthetized for 5 min in 0.02% Tricaine and eyeballs were extracted. Retinas were extracted from the eyeball and minced in 60 µl of 10 mM HEPES (pH 7.5), 110 mM NaCl, 2 mM CaCl_2_, 2.5 mM KCl and 1.2 mM MgCl_2_buffer on a glass slide using two 30-G needles. EGFP fluorescence in living cells was imaged immediately using an LSM 510 confocal microscope (Zeiss).

For cryosectioning, anesthetized tadpoles were fixed in 4% paraformaldehyde for 45 min, followed by incubation in 30% sucrose in PBS for 1 hr, and 30% sucrose and OCT (1∶1) solution for 1 hr. Tadpole heads were dissected and embedded in OCT and frozen at -80°C until use. Cryo-sections were made using Leica Microm Cryostat HM505E and stored at −80°C until use. Sections were incubated in 0.2% Triton in PBS for 30 min and stained with 2 µg/ml Alexa Fluor Wheat Germ Agglutinin 594 conjugates (Molecular Probes, Invitrogen, Carlsbad, CA) dissolved in 0.2% Triton-PBS for 30 min. Sections were washed twice with PBS containing 0.2% Triton and twice with PBS only. For nuclear counter-staining, sections were treated with 5 µg/ml RNAse A (Thermo Scientific) in PBS for 5 min and then with 1 µM TO-PRO3 (Invitrogen, Carlsbad, CA) solution in PBS for 10 min. Slides were washed briefly in PBS and mounted using Vectashield mounting medium (Vector Labs, Burlingame, CA). Extraction of EGFP-PDE6β1-313 and EGFP-PDE6β1-314fs*50 from transgenic retinas with isotonic and hypotonic buffers and immunoblotting of the extracts with anti-GFP antibody were performed as previously described [18].

### Expression and purification of PDE6β1-314fs*50 and PDE6β1-313

The 1098 bp cDNA encoding the human PDE6β1-314fs*50 mutant was synthesized (GENECUST-Europe, Luxembourg) and cloned into pFastBacHTb vector. Recombinant bacmids were generated to produce high-titer baculovirus stocks used to transfect Sf9 cells and express PDE6β1-314fs*50. PDE6β1-314fs*50 was solubilized in buffer with 6 M urea but when urea is removed completely, PDE6β1-314fs*50 precipitates. Some proteins stays in 2 M urea but 1–2 M urea already inhibits PDE6 activity.

DNA coding PDE6β1-313 was PCR amplified from the pXOP(−508/+41)EGFP-PDE6β1-313 plasmid and cloned into the pET15b vector using *Nde*I/*BamH*I sites. The His_6_-tagged PDE6β1-313 was expressed in BL21-codon plus *E. coli* cells by induction with 100 µM IPTG at 16°C overnight. Cell pellets were sonicated on ice (five 30-sec pulses) in 50 mM Tris-HCl buffer (pH 8.0) containing 50 mM NaCl, 5 mM MgSO_4_, 100 µM PMSF, 5 mM β-ME, 10 µM cGMP and EDTA-free protease inhibitor cocktail tablets (Roche). Lysates were centrifuged at 80000 g, 1 hr at 4°C. Supernatant was loaded onto Ni-NTA His-bind resin (Novagen) charged with 50 mM NiSO_4_ solution. Column was equilibrated or washed with 20 mM Tris-HCl (pH 7.5), 500 mM NaCl, 10 mM Imidazole and 20% glycerol. Finally, PDE6β1-313 was eluted with 20 mM Tris HCl buffer (pH 7.5), 250 mM NaCl, 200 mM Imidazole, 1 mM β-ME and 20% glycerol. 10 mM MgSO_4_ was added to the elutions and stored at 4°C until use.

### PDE6 activity assay

PDE6 activity was measured using holoPDE6 or trypsin-activated bovine PDE6 (tPDE6) and [^3^H]cGMP as a substrate. Hypotonic extract of bovine rod outer segment membranes containing holoPDE6 were prepared according to published protocols [19], [20]. tPDE6 lacking the inhibitory P**γ** subunits was prepared and purified as previously described [5]. HoloPDE (1 nM) or tPDE6 (50 pM) were incubated in 40 µL of 20 mM Tris-HCl (pH 7.5) buffer containing 50 mM NaCl, 1 mM MgSO_4_, 1 mM 2-mercaptoethanol, 0.1 U bacterial alkaline phosphatase, 0.1 mg/ml BSA, and 100 µM [^3^H]cGMP (30,000 cpm) at 25°C. After addition of [^3^H]cGMP, the reaction was allowed to proceed for 10 min and was stopped by the addition of AG1-X2 cation exchange resin (0.5 mL of 20% bed volume suspension). Samples were incubated for 6 min at 25°C with occasional mixing and spun at 9000 g for 3 min. Aliquots of 0.25 mL were removed for counting in a scintillation counter. To determine the IC_50_ values for the tPDE6 inhibition by Pγ in the presence or absence of 2 µM PDE6β1-313, PDE6 activity was measured with the additions of increasing concentrations of Pγ. Recombinant bovine rod Pγ-subunit was expressed in *E. coli* and purified as previously described [21]. The IC_50_ values were calculated by fitting data to sigmoidal dose-response equation Y(%) = 100/(1+10∧((X-LogIC_50_)), where *X* is the logarithm of total Pγ concentration. Fitting the experimental data to equations was performed with nonlinear least squares criteria using GraphPad Prizm Software.

### cGMP-binding assay

cGMP binding assay was performed in a total volume of 100 µl of 20 mM Tris-HCl (pH 7.5) buffer containing 100 mM NaCl, 2 mM 2-mercaptoethanol, 1 mM EDTA, 10 pmol of PDE6β1-313, [H^3^]cGMP (∼100,000 cpm) and varying concentration of unlabeled cGMP. The binding reactions were incubated for 2 hrs at 4°C and then applied onto pre-wet 0.45 µm cellulose nitrate membrane filters (Millipore). The filters were washed twice with1 ml of cold PBS buffer (pH 7.5) containing 1 mM EDTA, and counted by liquid scintillation counting. The data were fit to equation for binding with ligand depletion.[22]

### Fluorescence assay of PDE6β1-313 binding to Pγ24-45

Peptide Cys-Pγ24-45 was synthesized, purified, and labeled at the N-terminal Cys with the environmentally sensitive fluorescence probe 3-(bromoacetyl)-7-diethyl-aminocoumarin (BC) similarly as described previously [23]. Fluorescence measurements were performed on a F-2500 Fluorescence Spectrophotometer (Hitachi) in 1 ml of 100 mM PBS buffer (pH 7.5). Changes in fluorescence of CysBC-Pγ24-45 (50 nM) on addition of various concentrations of PDE6β1-313 were monitored with excitation at 445 nm and emission at 490 nm. The concentration of CysBC-Pγ24-45 was determined using ε_445_ = 53,000. The K_d_ value was calculated by fitting the data to the hyperbola equation: F = F_0_+F_max_*X/(K_d_+X) where F_o_ is a basal fluorescence of CysBC-Pγ24-45, F is the fluorescence after additions of PDE6β1-313, F_max_ is the maximal fluorescence, and X is a concentration of added PDE6β1-313.

## Results

### Clinical findings

**Figure 1:**
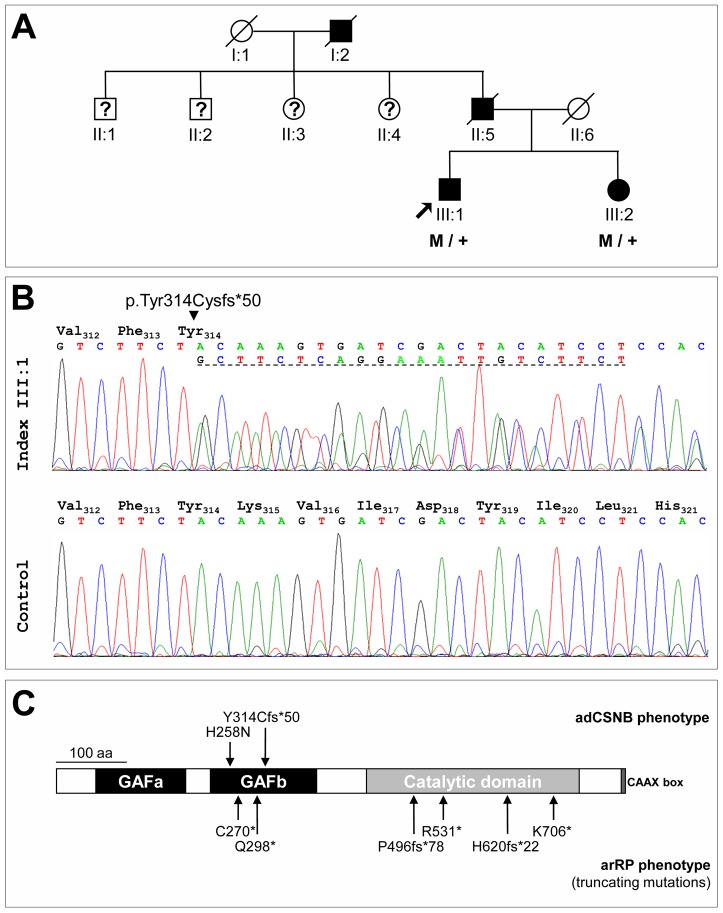
Pedigree with adCSNB and *PDE6B* mutation. A. The three-generation French family MTP1481 with adCSNB. Filled symbols indicate affected family members; squares: males; circles: females; arrow: index patient; slashed symbols: deceased persons; question marks: family members with no information about the phenotype available. M indicates the presence of the mutation and + indicate the wild-type allele. B. Electropherograms show the normal control sequence and affected sequence (index individual III:1) surrounding the p.Tyr314Cysfs*50 mutation. The dotted line represents the 22 duplicated nucleotides. C. Schematic representation of the PDE6β protein showing the domain structure and positions of arRP truncating mutations (below) and adCSNB mutations (above) including the mutation described in this study (Y314Cfs*50).

**Figure 2:**
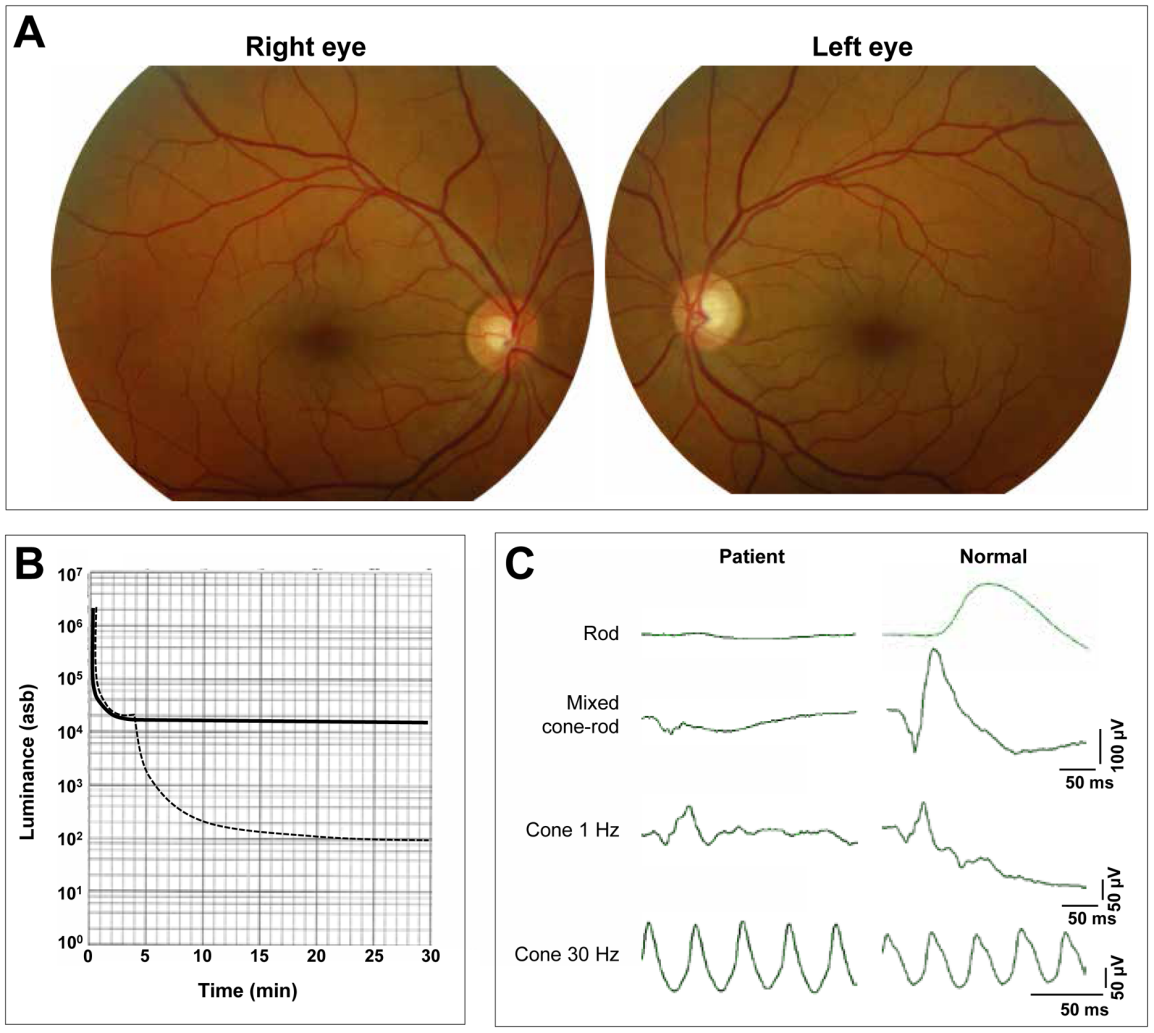
Clinical investigations of the index patient (III:1) of a French family with adCSNB. A. Fundus photographs of index patient III:1, right eye and left eye. B. Dark-adaptation curve of index patient III:1 (solid line) compared to normal curve (hatched line). D. Full-field ERGs from a normal control and index patient III:1.

The three-generation French family MTP1481 presented typical congenital stationary night blindness (CSNB) symptoms segregating as an autosomal dominant trait (Figure 1A). The index patient (III:1) and his sister (III:2) complained about non-progressive night blindness with normal day vision since early childhood. They had neither visual field loss nor photophobia and their reading capacity was normal. At presentation, the 40 year-old index subject had normal visual acuity at 1.5 on both eyes. Anterior segments were normal and the lenses were transparent. The fundus showed no sign of retinal degeneration as the posterior pole, macula, and optic discs had normal appearance, and the retinal vessels were not attenuated (Figure 2A). Dark adaptometry revealed normal cone dark adaptation in 4 min but total absence of rod adaptation, with an absolute threshold remaining at 2 log units above the normal level at 30 min (Figure 2B). The electroretinogram showed the absence of rod responses while the cone responses were normal (Figure 2C). In addition, there was an electronegative waveform (absence of b-wave) at mixed rod cone stimulation, typical of stationary night blindness (Figure 2C). The sister subject III:2 was not available for an ophthalmic examination.

### Identification of a truncating mutation in PDE6B

Direct sequencing of the three known genes responsible for autosomal dominant CSNB (adCSNB) was performed in the index patient and no mutation was found for *RHO* and *GNAT1*, except an intronic variant in *RHO* (NM_000539.3), c.361+10G>A, absent in the affected sister and found in the EVS database at a low frequency (1/13006 alleles). However, the duplication c.928-9_940dup was identified in the *PDE6B* (NM_000283.3; Figure 1B). This variation was not present in the public human SNP databases (including dbSNP, Ensembl, HapMap, the 1000 Genomes project and Exome Variant Server). This insertion of 22 nucleotides in exon 6 caused a tyrosine to cysteine substitution at codon 314, and led to a frameshift and a premature termination, shortening the protein to 362 residues instead of 854 amino acids (p.Tyr314Cysfs*50). This novel mutation was an insertion of the sequence GCTTCTCAGGAAATTGTCTTCT between the nucleotides 940 and 941 (c.940_941insGCTTCTCAGGAAATTGTCTTCT). His affected sister (III:2) was also heterozygous for the mutation (Figure 1A). The DNA of the father and grandfather were not available for sequencing.

### PDE6β1-313 binds the polycationic region of Pγ, noncatalytic cGMP, and interferes with the Pγ inhibition of PDE6

**Figure 3:**
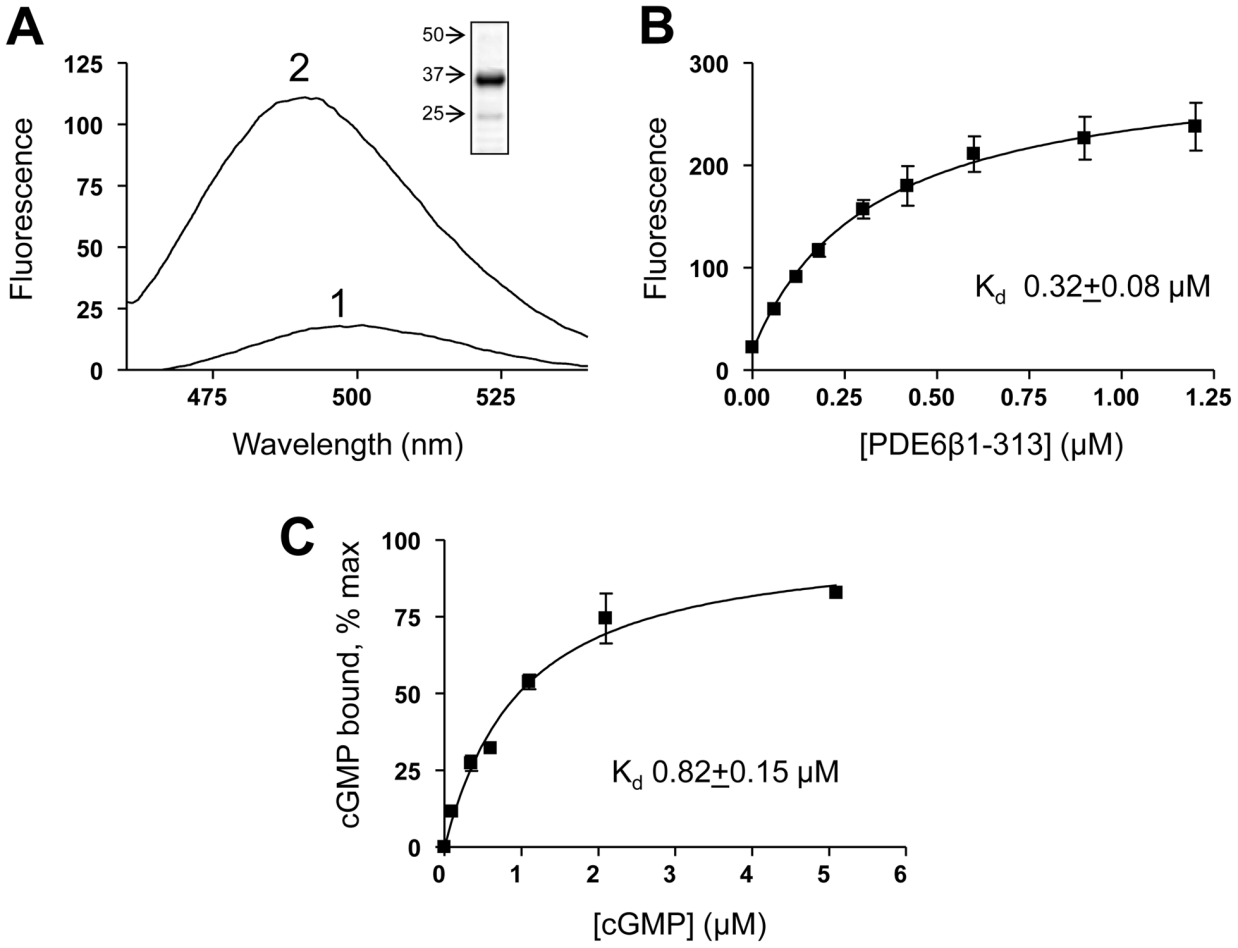
Interaction of PDE6β1-313 with the polycationic region of Pγ, Pγ24-45. A. Emission spectra of CysBC-Pγ24-45 (50 nM) alone (1) and in the presence of PDE6β1-313 (180 nM) (2) were recorded with with excitation at 445 nm. Inset: Coomassie Blue-stained gel with the sample of PDE6β1-313. B. Fluorescence of CysBC-Pγ24-45 (50 nM) was measured on addition of increasing concentrations of PDE6β1-313 (excitation at 445 nm, emission at 490 nm). The data were fit to the hyperbola equation using GraphPad Prizm software. C. Noncatalytic cGMP binding to PDE6β1-313. Binding of cGMP was carried out using [H^3^]cGMP and varying concentrations of unlabeled cGMP. The bound cGMP was determined by the filter-binding assay, and is expressed as a percentage of maximal cGMP binding (0.5 mol cGMP/mol PDE6β1-313). Results from one of three similar experiments are shown. The data were fit to equation for binding with ligand depletion using GraphPad Prizm software. The calculated K_d_ = 0.82±0.15 µM.

The insertion mutation in *PDE6B* is predicted to result in expression of the 313 amino acids of the N-terminal portion of PDE6β protein with the artificial 50-residue long adduct due to the frameshift. We have attempted to express the PDE6β1-314fs*50 protein product of the mutated gene using the baculovirus/sf9 cell system. However, the recombinant protein was unstable and precipitated out of solution during purification. In contrast, expression of the PDE6β specific portion of the mutant, PDE6β1-313, in *E. coli* yielded high levels of soluble protein (Figure 3A, inset). The PDE6β1-313 sequence contains the GAFa domain and a portion of the GAFb domain (Figure 1C). The GAFa-domains of PDE6 have been shown to bind noncatalytic cGMP [24], [25] and interact with the polycationic region of Pγ, Pγ24-45 [26]. The possible binding of Pγ24-45 to PDE6β1-313 was investigated using the fluorescence binding assay. Addition of PDE6β1-313 to CysPγ24-45 labeled with the environmentally sensitive fluorescence probe, CysBC-Pγ24-45, caused a large dose-dependent increase in the probe fluorescence (Figure 3AB). The binding was specific as the fluorescence increase was blocked by excess of unlabeled CysPγ24-45 (not shown). The K_d_ of 0.32 µM calculated from the binding curve suggested a relatively strong interaction between PDE6β1-313 and Pγ24-45 (Figure 3B). We next examined if PDE6β1-313 is capable of binding cGMP. The cGMP-binding assay revealed a single class of cGMP-binding sites on PDE6β1-313 with the affinity (K_d_ = 0.82 µM) comparable to that of cGMP binding by the GAFa domain of cone PDE6 (Figure 3C).[27]

**Figure 4:**
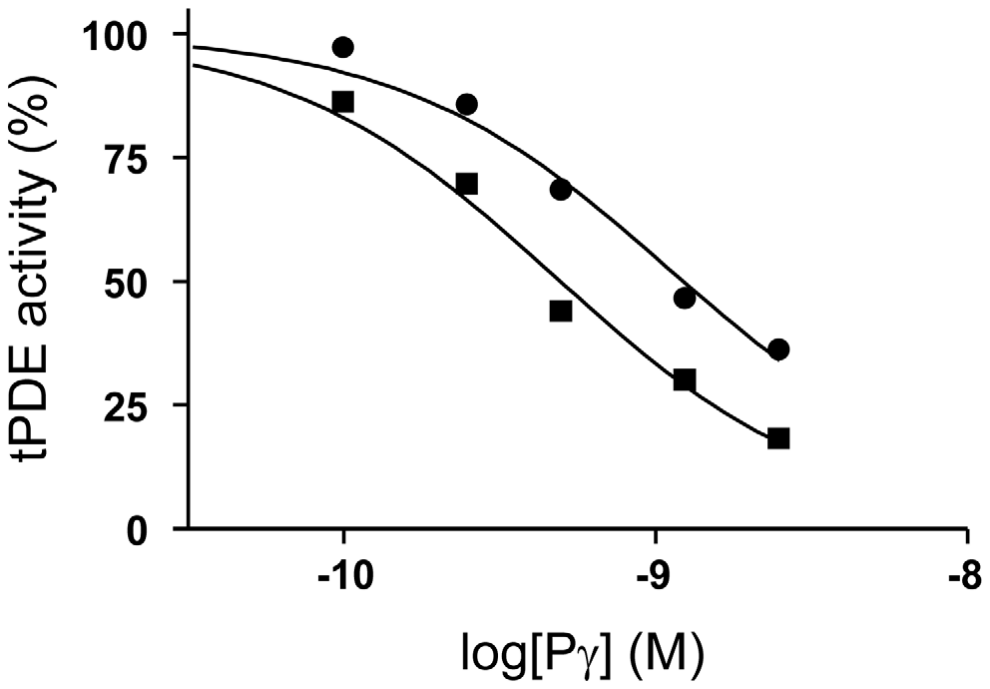
Effect of PDE6β1-313 on the inhibition of trypsin-activated PDE6 (tPDE6) by Pγ. The activity of tPDE6 was determined upon addition of increasing concentrations of Pγ in the absence (squares) or in the presence (circles) of 2 µM PDE6β1-313. The tPDE6 activity is plotted as a function of Pγ concentration. Results from one of three similar experiments are shown. The data were fit to sigmoidal dose-response equation using GraphPad Prizm Software. The calculated IC_50_ values (squares, 0.53±0.07 nM; circles, 1.6±0.2 nM) are significantly different (p<0.01).

The interaction of PDE6β1-313 with the polycationic region of Pγ indicated that PDE6β1-313 may interfere with the ability of Pγ to inhibit normal PDE6αβ. We tested this hypothesis by measuring the effects of PDE6β1-313 on basal activity of holoPDE6 and on inhibition of trypsin-activated bovine PDE6 (tPDE6) by Pγ. PDE6β1-313 had no effect on either the basal activity of holoPDE6 or on the tPDE6 activity (not shown). However, the potency of tPDE6 inhibition by Pγ was notably decreased in the presence of PDE6β1-313 (Figure 4). This effect cannot be explained by sequestration of cGMP by PDE6β1-313 since the concentration of cGMP in the assay was much higher than that of PDE6β1-313.

### PDE6β1-313 traffics to the rod outer segment

**Figure 5:**
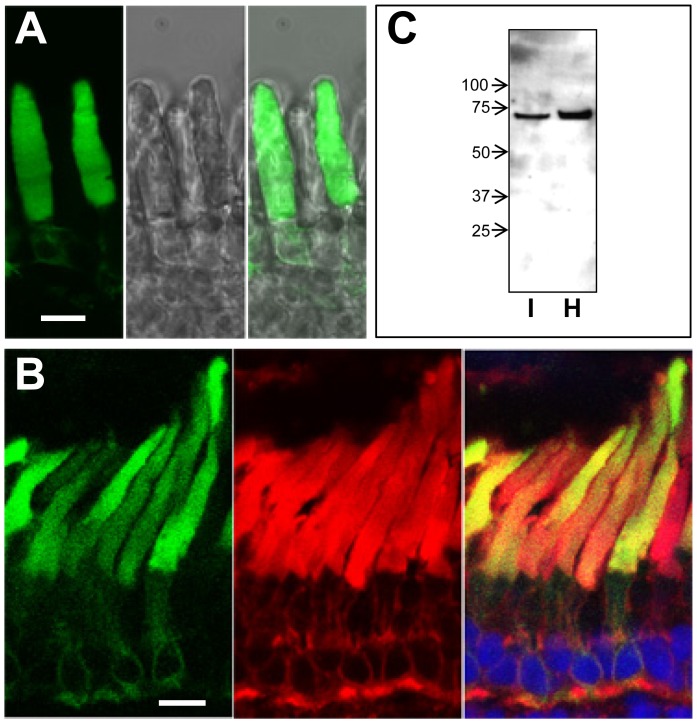
Expression of PDE6β1-314fs*50 in transgenic *X. laevis* rods photoreceptors. A. *left* - EGFP fluorescence in living photoreceptor cells expressing PDE6β1-314fs*50, *center* - DIC image, *right* - EGFP-fluorescence/DIC overlay. *Bar* - 10 µm. B. Co-localization of PDE6β1-314fs*50 (green) with the rod outer segment marker Wheat Germ Agglutinin (red) in cryosections of transgenic retina. Cryosections were counterstained with TO-PRO3 nuclear stain (blue). *Bar* - 10 µm. C. Isotonic (I), hypotonic (H) extracts of transgenic PDE6β1-314fs*50 retinas were analyzed by immunoblotting with anti-GFP B-2 monoclonal antibody.

**Figure 6:**
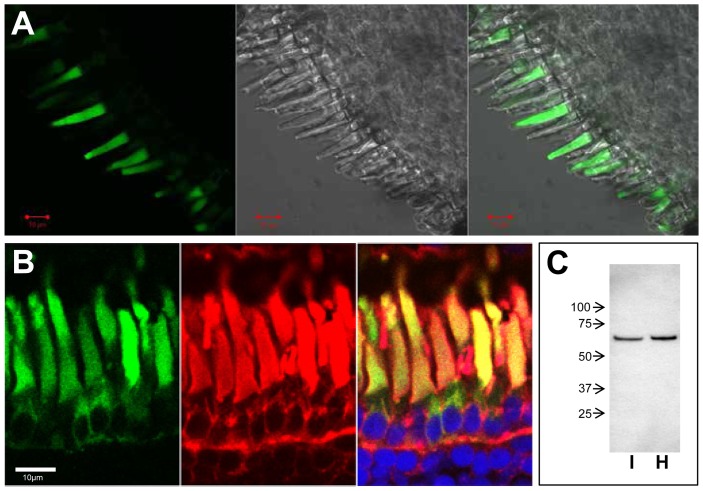
Expression of EGFP-PDE6β1-313 in transgenic *X. laevis* rods photoreceptors. A. *left* - EGFP fluorescence in living photoreceptor cells expressing EGFP-PDE6β1-313, *center* - DIC image, *right* - EGFP-fluorescence/DIC overlay. *Bar* - 10 µm. B. Co-localization of EGFP-PDE6β1-313 (green) with the rod outer segment marker Wheat Germ Agglutinin (red) in cryosections of transgenic retina. Cryosections were counterstained with TO-PRO3 nuclear stain (blue). *Bar* - 10 µm. C. Isotonic (I), hypotonic (H) extracts of transgenic EGFP-PDE6β1-313 retinas were analyzed by immunoblotting with anti-GFP B-2 monoclonal antibody.

To determine the localization of PDE6β1-314fs*50 and PDE6β1-313 in rods, and thus, the probable site of action of the PDE6β truncation mutant, we generated transgenic *X. laevis* expressing EGFP-fused PDE6β1-314fs*50 and PDE6β1-313 in rod photoreceptors (Figures 5, 6). Live EGFP-fluorescence imaging of transgenic *X. laevis* retina indicated that both PDE6β1-314fs*50 and EGFP-PDE6β1-313 are targeted to the rod outer segments (OS) (Figure 5A, 6A). Similarly, the imaging of retina cryosections also showed the major fractions of PDE6β1-314fs*50 and EGFP-PDE6β1-313 in the OS (Figure 5B, 6B). Interestingly, the diffuse patterns of PDE6β1-314fs*50 and EGFP-PDE6β1-313 in the OS are different from the previously described striated and peripheral pattern of EGFP-PDE6C [16]. The targeting of PDE6β1-314fs*50 to the OS, suggests that, in contrast to its misfolding in insect sf9 cells, this truncated PDE6β protein is folded when expressed in rod photoreceptors. Immunoblot analyses of retinal extracts from transgenic tadpoles with anti-GFP antibody showed that PDE6β1-314fs*50 and PDE6β1-313 were partially membrane-bound in isotonic buffer, since significant fractions of the proteins were subsequently solubilized in hypotonic buffer (Fig. 5C, 6C). Thus, PDE6β1-314fs*50 and PDE6β1-313 lacking lipid modifications retain the ability to associate with intracellular membranes (Fig. 5C,6C).

## Discussion

Six genes encoding major components of the phototransduction cascade (*RHO*, *GNAT1*, *PDE6B, GRK1*, and *SAG*) and the regeneration of the visual pigment (*RDH5*) are described in congenital stationary night blindness (CSNB) patients. Among these, *RHO*, *GNAT1* and *PDE6B* encode the rhodopsin, the rod transducin α-subunit (Gtα), and the rod phosphodiesterase-6 β-subunit (PDE6β) respectively and cause the autosomal dominant form of the disease (adCSNB). Only seven mutations in *RHO*, *GNAT1* and *PDE6B* are described in adCSNB patients. Among these, four *RHO* mutations (p.Gly90Asp, p.Thr94Ile, p.Ala292Glu, and p.Ala295Val) [2], [28]–[30], one single *PDE6B* mutation (p.His258Asn) [4], and one *GNAT1* mutation (p.Gln200Glu) [31] result in constitutively active proteins. While another *GNAT1* mutation (p.Gly38Asp) [3] results in Gtα unable to interact with its downstream effector PDE6 *in vitro* [32], the mutant Gtα can apparently activate PDE6 with reduced efficiency *in vivo* [33]. Thus, GtαGly38Asp may cause adCSNB due to its defective GTPase activity and inactivation [33]. A third mutation in *GNAT1* (p.Asp129Gly) was recently described in arCSNB patients [34]. Mutations in *RHO* and *PDE6B*, but not *GNAT1*, are also described in retinitis pigmentosa (RP). However, one *RHO* missense mutation (p.Gly90Asp) was observed in patients with either adRP or adCSNB [35]. Therefore, in most if not all cases, the constitutive activation of the phototransduction cascade is a prerequisite to cause adCSNB.

Currently, 43 different mutations in *PDE6B* are listed in the human genome mutation database (HGMD) that lead to autosomal recessive RP (arRP). These mutations lead to a complete loss of PDE6 enzymatic activity and the resulting increase of the intracellular cGMP level is the cause of the rod degeneration [36]–[40]. Elevated levels of cGMP lead to toxicity followed by apoptosis in rod photoreceptors [39], [41]. In contrast, the only known stationary rod dysfunction adCSNB-associated *PDE6B* change found in the Rambusch pedigree, the missense substitution p.His258Asn (H258N), leads to rod desensitization of dark-adapted photoreceptors [4]. The PDE6βH258N substitution appears to alter, rather than abolish, the rod PDE6 function [42]. The study indicated that the PDE6βH258N mutant has an impaired interaction with Pγ [42]. Incomplete inhibition of PDE6αβ by Pγ in the dark would result in constitutive PDE6 activity leading to desensitization of rod photoreceptors and to night blindness. The CSNB night blindness is similar to the early stage of RP, but without the progressive impairment of the day vision. In this study, we report a second mutation for this condition and functional analysis of the mutated PDE6β.

The DNA of the index patient of the French family MTP1481 (Figure 1A), diagnosed with adCSNB, was screened for mutation in the three known genes responsible for this condition (*RHO*, *GNAT1* and *PDE6B*). Although the family is originating in the south of France, the Nougaret type mutation, p.Gly38Asp, was not found in *GNAT1*. A novel mutation, the duplication c.928-9_940dup, was identified in the *PDE6B* gene (Figure 1B), which results in a tyrosine to cysteine substitution at codon 314, a frameshift, and a premature termination shortening the protein to 362 residues instead of 854 amino acids (p.Tyr314Cysfs*50). The mutation located within the second GAF domain (GAFb) truncates the protein upstream from the catalytic domain and the C-terminal isoprenylation site but does not affect the first GAF domain (GAFa) containing the Pγ binding site (Figure 1C). Thus, the resulting mutated protein is predicted to lack the enzymatic activity. However, if properly folded, the mutant may still be able to bind Pγ. We hypothesized that, in order to produce a dominant effect on phototransduction, the truncated PDE6β mutant is expressed as a folded protein, which is targeted to the phototransduction compartment where it causes constitutive PDE6 activity by interacting with Pγ.

To test our hypothesis, we examined localization of the PDE6β1-314fs*50 and EGFP-PDE6β1-313 proteins expressed in rods of transgenic *X. laevis*. In addition we investigated the biochemical properties of the bacterially expressed PDE6β1-313. The immunoblot analysis of transgenic *X. laevis* retina extracts showed bands for PDE6β1-314fs*50 and EGFP-PDE6β1-313 of appropriate size with no signs of proteolytic degradation (Figure 5C, 6C). The imaging of transgenic frogs demonstrated that PDE6β1-314fs*50 and EGFP-PDE6β1-313 are trafficking to the rod outer segment (Figure 5, 6). This result is in agreement with the recent findings that the PDE6 GAFa domain contains an OS targeting motif [18]. PDE6β1-314fs*50 lacks the lipid modifications of native PDE6 essential for trafficking of the enzyme in rods [43]. Nonetheless, we found that PDE6β1-314fs*50 and PDE6β1-313 are partially membrane-bound in isotonic solutions (Fig. 5C, 6C). The membrane affinity of the truncated PDE6β form is likely to be involved in its trafficking to the membrane-rich OS compartment.

The expression of soluble PDE6β1-313 in *E. coli* was robust. Recombinant PDE6β1-313 bound noncatalytic cGMP supporting correct folding of the protein. This is in agreement with the previous studies, showing that the individual GAFa domain of PDE6C can be expressed as a soluble and properly folded protein that binds cGMP [25]. Furthermore, recombinant PDE6β1-313 potently bound the fluorescently labeled Pγ24-45 (K_d_ 0.32 µM), confirming our supposition that the GAFa domain in the truncated mutant is capable of binding the polycationic region of Pγ (Figure 3). This K_d_ is only about one order of magnitude higher than the K_d_ value determined previously for binding of Pγ24-45 to native PDE6αβ [23]. We further show that while PDE6β1-313 has no significant effect on the basal activity of holoPDE6, it interferes with the Pγ inhibition of trypsin-activated PDE6 (Figure 4). The latter effect is presumably due to competition of PDE6β1-313 with the full-length PDE6αβ for binding to Pγ. We were unable to investigate the properties of the exact mutant gene product in humans, PDE6β1-314fs*50, due to instability of the recombinant protein *in vitro*. Nonetheless, we surmise that PDE6β1-313 is reflective of the truncated human PDE6β mutant. The effect of PDE6β1-313 *in vitro* is not potent and requires micromolar concentrations of the protein compared to subnanomolar Pγ necessary for PDE6 inhibition (Fig. 4). In rods, light activation of a fraction of total PDE6 is sufficient to saturate the signaling. Moreover, an incomplete inactivation of only a fraction of activated PDE6 may lead to strong desensitization. Therefore, PDE6β1-314fs*50 at micromolar concentrations (if expressed comparably to native PDE6) would be competing with activated (incompletely inactivated) PDE6 present at much lower concentrations. Meaningful competition is plausible under these conditions. Accordingly, we propose that following light stimulation in a human patient, the truncated PDE6β mutant is likely to prevent complete inactivation of transducin-activated PDE6 leading to prolonged or constitutive activity. Thus, our results support the hypothesis that the truncated PDE6β mutant constitutively activates the phototransduction cascade and causes the desensitization of rod photoreceptors.

The general cause of adCSNB due to constitutive PDE6 activity appears to be similar in the case of the truncated PDE6β mutant and the Rambusch form with the PDE6β substitution p.His258Asn [4]. However, the mechanistic basis for the persistent PDE6 activity might be different between the two mutant forms. The p.His258Asn mutation is a cis-acting mutation which causes incomplete inhibition of PDE6αβ by Pγ [42]. Therefore, the basal PDE6 activity is expected to be elevated in the Rambusch form. The truncated PDE6β mutant may rather be regarded as trans-acting which may cause incomplete or delayed inactivation of PDE6 following activation of phototransduction.

Among the 39 arRP-associated *PDE6B* mutations, several are premature termination codon resulting from nonsense or frameshift mutations (Figure 1C). The patients with a nonsense mutation are either homozygous (p.Cys270* [44]) or compound heterozygous (p.Gln298* and p.Arg531* [14]; p.Lys706* and p.Gln298* [45]) with two nonsense mutations. The patients with a frameshift mutation are compound heterozygous with the frameshift mutation and a missense mutation (p.Pro496fs*78 and p.His557Tyr [14]; p.His620Glnfs*22 and p.Gly576Asp [46]). Contrary to the mutation described in this study, these truncating mutations are causing arRP and not adCSNB. The different phenotypes could be explained by the nonsense-mediated mRNA decay (NMD), a physiological surveillance mechanism. Aberrant mRNA harboring a premature nonsense codon is degraded by NMD, and thus the synthesis of abnormal proteins is eliminated [47], [48]. We hypothesized that the p.Tyr314Cysfs*50 frameshift mutation results in the production of the truncated protein, rather than in the decreased abundance of affected mRNA transcripts to explain the dominant effect in the family MTP1481. There is some difference in the efficiency of NMD for transcripts with premature termination codon resulting from different frameshift mutations [47]. We can imagine the arRP frameshift mutations, which are located in the catalytic domain, are either eliminated or expressed, and in the latter case, there is no dominant effect.

In conclusion, we have identified a novel adCSNB-causing mutation, c.928-9_940dup (p.Tyr314Cysfs*50), in *PDE6B*. Our study indicates that this truncated mutation, unable to hydrolyze cGMP, apparently causes constitutive activation of the phototransduction through its ability to bind the inhibitory Pγ subunit, and hence rod desensitization.

### Web Resources

dbSNP, http://www.ncbi.nlm.nih.gov.gate2.inist.fr/SNP/

Ensembl, http://www.ensembl.org/index.html

EVS, http://evs.gs.washington.edu/EVS/

HapMap, http://hapmap.ncbi.nlm.nih.gov/

HGMD, http://www.hgmd.cf.ac.uk/ac/index.php

OMIM, http://www.omim.org

1000 genomes project, http://browser.1000genomes.org/index.html
